# Exploring how Professional Associations Influence Health System Transformation: The Case of Ontario Health Teams

**DOI:** 10.5334/ijic.7017

**Published:** 2023-05-24

**Authors:** Alyssa Indar, James Wright, Michelle Nelson

**Affiliations:** 1 Institute of Health Policy, Management and Evaluation, University of Toronto, Toronto, Canada; 2 Economics, Policy and Research Division, Ontario Medical Association, Toronto, Canada; 3 Lunenfeld-Tanenbaum Research Institute/Sinai Health System, Toronto, Canada

**Keywords:** qualitative research, professional associations, Ontario Health Teams, health system transformation

## Abstract

**Introduction:**

Health care system transformations that align with the principles of integrated care require the collaborative efforts of various macro-, meso- and micro-level stakeholders. Understanding the roles of various system actors can improve collaboration in ways that support purposeful health system change. Professional associations (PAs) have considerable influence, but little is known about the strategies they use to influence health system transformation.

**Methods:**

Using a qualitative descriptive approach, eight interviews with 11 senior level leaders from local PAs were conducted to learn about the strategies used to influence the province-wide reorganization of health care into Ontario Health Teams.

**Results:**

During times of health system transformation, PAs balance: (1) supporting members, (2) negotiating with government, (3) collaborating with stakeholders, and (4) reflecting on their role. The enactment of these various functions demonstrates the strategic nature of PAs, and showcases their ability to evolve in ways that align with the dynamic nature of healthcare.

**Discussion:**

PAs are highly connected groups, deeply engaged with their members and regularly engaged with other key stakeholders and decision-makers. PAs play a critical role in influencing health system transformations, by bringing forward practical solutions to government that reflect the needs of their members, often frontline clinicians. PAs strategically seek opportunities for collaboration with stakeholders that can amplify their message.

**Conclusion:**

Insights from this work could support health system leaders, policymakers, and researchers in leveraging the role of PAs in health system transformations via strategic collaboration.

## Introduction

Globally, health systems are increasingly undergoing large scale transformations that align with the principles of integrated care, to improve the delivery of high quality, coordinated and patient-centred care [1,2]. Health system transformation requires the collaborative efforts of a large swath of macro-, meso- and micro-level actors, which often include government, professional associations, organizations, health care providers and many others [3]. Recent literature highlights the need for leaders to look beyond their organization’s interests in order to seek opportunities to build relationships with stakeholders that can improve local collaboration [4]. Although there is evidence to guide intersectoral and inter-organizational collaboration during health care system transformations, the specific role of professional associations (PAs) as stakeholders with considerable influence in health and social care, has remained largely unexplored within this area. PAs vary considerably in size and specific mandate, but primarily serve to advance the interests of their membership [5]. Many PAs represent specific health professions; a large well-known example is the American Medical Association, which advocates on behalf of American physicians, particularly in the area of health policy by influencing legislation [6].

From the literature, it is known that PAs represent the interests of their membership and play key roles in influencing health policy which impact health care planning and delivery [7]; however, there is no ‘playbook’ to guide their strategies or actions when interacting with other stakeholders, government, and the public [8]. Although most PAs have clear mandates to advance the interests of their members, PAs must also enact strategic thinking to strike the ‘right’ balance to satisfy the interests of many stakeholders [9]. Enacting these skills are critical amid the uncertainty of health system transformation when there may be a heightened need to weigh the interests and needs of their individual members against the collective needs of the health system [10]. In cases where member needs and system needs are not aligned, PAs face difficult decisions when choosing a formal position and a corresponding tactic. There is limited scholarly research on the role and activities of PAs, perhaps due to the broad diversity in their structures and functions; and the private nature of their operations [7]. It is important to understand how PAs function, due to their high degree of influence in the health policy domain, which often shapes health care systems.

The development of Ontario Health Teams (OHTs) is a current example of a large-scale health system transformation that commenced in February 2019. This provided an opportunity to explore the broader phenomenon of how PAs influence health system transformations. The OHT strategy shares common characteristics with other integrated care programs described in international literature, as there are changes to service organization, funding and care delivery in ways that align with the principles of patient-centred care [11]. OHT development is supported by government-driven health care reform and is intended to bring together health and social services in ways that serve patients and families. Historically, Ontario has undergone several iterations of health system restructuring in the past few decades to improve system integration, such as the development of Local Health Integration Networks and Health Links [12,13].

The current vision for OHT development is to enhance care coordination by reorganizing existing health and social care services into approximately 51 OHTs that will provide care for 95% of patients in Ontario [14]. This strategy is underpinned by a population health approach, in which OHTs, comprised of local health organizations and providers, will each implement a model of care that best suits the needs of their priority patient population [15]. The rollout of OHTs has been described as “low rules”, due to the minimal involvement and funding from the government [16]. In essence, each OHT will be allowed to customize care for their patient population, by creating new governance structures that enable a high level of collaboration between health and social care sectors. The process for forming OHTs is outlined by the provincial Ministry of Health (MOH). The initial phase required prospective OHT members to establish a collaborative decision-making arrangement (CDMA) to support inter-organizational, consensus-oriented decision making based on shared goals and accountabilities [17]. It was expected that CDMAs address concerns related to “resource allocation, information sharing, financial management, inter-team performance discussion, dispute resolution, conflicts of interest, quality improvement” and related topics [17]. Therefore, there were significant contractual and financial implications for these members.

PAs are not considered members of OHTs; therefore, there are no such direct implications for them. However, PAs within Ontario have been active in OHT development, given the wide-ranging impact of the system changes on their clinician members. For example, PAs may be responding to an influx of member concerns, ranging from individual concerns (e.g., compensation) to questions regarding how the system level changes could change their everyday work environments. Clinician perspectives are important to consider, as clinician engagement and effective management of health human resources are important factors for success in health system transformations [10,13]. A critical literature review indicated that PAs may be leveraging a variety of strategies during times of health system transformation to advance their members’ interests; these strategies involve communicating with the government, public and other relevant stakeholders [8]. The aim of this qualitative descriptive research study was to explore how PAs influence health system transformation, focusing on OHT development as a tangible example of health system transformation. This was accomplished by interviewing senior leaders from eight PAs, that were involved in OHT development, to learn more about how and when specific strategies were used to directly and/or indirectly influence the development of OHTs.

It is important to acknowledge the impact of the COVID-19 pandemic on the rollout of OHTs. In March 2020, Ontario experienced the first COVID-related lockdown, or restriction of access to non-essential activities. In the following months, there was enormous strain on the health care system, with emphasis on issues related to overwhelmed intensive care units and resident deaths in long-term care [18]. The immediate need to stabilize the delivery of health care slowed and, in a few cases, accelerated the development of OHTs. However, the COVID-19 pandemic emphasized the need for building a more integrated and coordinated approach to health care in Ontario.

## Methods

This project was co-funded by the Canadian Institutes of Health Research (CIHR) and the Ontario Medical Association (OMA), to support the work of a Health System Impact Fellow (AI). The fellowship aims to support graduate students in applying health services and policy research skills outside of traditional scholarly settings [19]. The fellow worked with a host organization, the OMA, to co-design a research proposal, relevant to the organization’s interests and that utilized the fellow’s research skillset. The OMA provided the fellow with access to a supportive learning environment and access to in-house resources. All research was conducted arms-length from the OMA. We received Research Ethics Board approval from the University of Toronto to conduct this research study (#42049).

### Participant Selection & Recruitment

To identify the PAs that have had the most involvement with (and effect on) OHT development, we conducted a grey literature review coupled with an expert consultation process. The grey literature review was supported by a team of librarians, with expertise in conducting searches related to PA content (e.g., media, websites). The librarian team identified the PAs with formal position statements on the topic of OHTs. This generated a list of PAs. Our research team shared the list of PAs with the OMA Stakeholder Team, for the purpose of receiving feedback. The Stakeholder Team manages communications with members, government, and other major stakeholders, which qualified them to provide this type of guidance. The Stakeholder Team helped us identify which PAs were most active in the realm of OHT development, which affirmed some of our selections and helped us to include PAs that we had not initially included. Ultimately, 10 PAs met the inclusion criteria of: (1) representing a group of members impacted by OHT development, and (2) being actively engaged in public discussion related to OHT development (e.g., publishing position statements, releasing media statements, etc.).

Due to the wide variation in size and structure of each PA, it was initially difficult to determine the most appropriate individual within each organization to interview. Therefore, we sent the initial round of recruitment emails to the CEO and/or President of each PA and invited them to either take part in an interview or delegate the interview to an individual in their organization with the appropriate level of knowledge. The inclusion criteria specified that all participants must (1) be an employee of one of the selected PAs, and (2) hold a leadership position with a level of knowledge sufficient to participate in an interview. Ultimately, 11 individuals from eight different PAs consented to participate in this research study. To protect the anonymity of our participants, all data shared in this report has been de-identified and anonymized.

### Data Collection

A qualitative descriptive approach [20] is helpful when seeking to generate descriptive themes that elucidate the selected phenomenon. Data were collected via semi-structured interviews with senior leaders from eight PAs. The interview guide was developed by all research team members and questions were grouped into the following sections: (1) description of PA structure and function, (2) strategies used to align member needs and system needs, (3) strategies used to influence OHT development, (4) stakeholder collaboration, and (5) government relations. The interview guide was piloted with three individuals, selected due to their current experience working in a PA. These individuals provided verbal feedback to enhance the clarity and logical flow of the interview questions.

Eight interviews were conducted, which represent perspectives from eight PAs. Most interviews were conducted with a single participant, but we accommodated requests to have multiple participants interviewed together, especially if their roles were complementary within their organization. Of the eight interviews, one was conducted with two participants, and another was conducted with three participants. Therefore, we interviewed a total of 11 participants in eight separate interviews. Each interview was 40–50 minutes in length and audio recorded. Data were transcribed and de-identified by AI. The interviews took place from February 2022 to June 2022. In consultation with our local Research Ethics Board, we opted not to report participant demographic information due to the risk of identifying them.

Undertaking this research study during the COVID-19 pandemic prompted our team to anticipate challenges and develop mitigation strategies. Firstly, all aspects of recruitment and data collection were virtual, which allowed for these processes to be minimally impacted if there were changes in pandemic restrictions. Secondly, we anticipated that the data collection period might be longer than expected, as PAs were managing higher workloads than usual. Therefore, we were able to collect data over a period of five months, which provided participants with increased flexibility when scheduling the interview.

### Data Analysis

Data were analyzed inductively using a content analysis approach, which consists of three main phases and aids in constructing themes (or categories) that describe the selected phenomenon [21]. In the first phase, we became immersed in the data by reading and re-reading the transcripts, discussing general impressions, and planning for next steps in the data analysis process. In the organizing phase, we engaged in open coding, grouped codes together and generated a code book. We used a Microsoft Word document to organize codes into larger categories and engaged in team discussion until we agreed on the organization of data into categories and subsequently into themes. Consistently engaging in team dialogue strengthened our reflexivity during the analysis process.

## Results

The study findings have been organized into four descriptive themes: (1) supporting members, (2) negotiating with government, (3) collaborating with stakeholders, and (4) reflecting on the PA role. These themes emphasize the connected nature of PAs, in which they frequently communicate with various stakeholders in the health policy ecosystem.

### Theme #1: Supporting members

All participants described the importance of maintaining regular communication with members, to keep them informed of changes related to OHTs or to elicit their feedback on these topics. Most PAs regularly surveyed their members to understand which issues were most important. For some PAs, it was sufficient to use this information to rank concerns and proceed with advocating for the ones that most members highlighted. Other PAs had elaborate processes to learn more about the issues highlighted by members, discuss the issues among senior leaders and explore tactics for effective advocacy.

Many participants emphasized the importance (and challenge) of seeking alignment between members – before considering how membership may be aligned with other system stakeholders.

“The challenge is less about the membership being aligned with broader system goals and more about the membership actually being aligned with each other.” (PA #5)

Although some member concerns would underpin advocacy efforts, some participants described the nuanced role of PAs in helping members to better understand components of the health system changes, to alleviate fears and to further aid in aligning member interests:

“[there was] myth busting and education, also helping members to understand the policy development moving towards implementation process. And key is trying to educate and hear from members on what is important to them.” (PA #8)

A few participants described the need to support members differently during times of system transformation. One participant suggested that PAs shift from being a ‘cheerleader’ to a ‘coach’, which emphasized their strategic influence:

“Sometimes we struggle with, are we a cheerleader for them or are we the coach? It’s always a constant balancing act as an association. We have to meet our member needs, but we also have to sort of lead from behind and because we see the overarching kind of strategic directions of the province, or the Ministry, or other stakeholders.” (PA #1)

Building on this, some participants described how their PAs strategically shifted modes of member engagement as OHT development progressed. Many of the following examples highlight the role of PAs in providing practical guidance for members at times when the vision for health system transformation is unclear:

“We did spend a lot of time especially early on…trying to get any engagement. In the beginning, we sent out letters to all the [members] in the province, just to say this is your OHT…we got a huge response. Some positive and some negative feedback…I think we did put a lot of effort into trying to support them. So they could support others”. (PA #8)

“[it’s] been a fundamental shift in our organization…we’re really trying to harness the leaders. We’re trying to harness those high performing teams and invest in them and work with them…we need to encourage the leadership and really spend our time and attention to sustain those high performing high functioning teams and learn from them. But spend less time on the lower performing teams.” (PA #1)

This theme underscores the high degree of importance that all participants placed on engaging with their membership. Many participants highlighted the nuanced differences in providing support during times of health care transformation. These PAs played important roles in “myth busting” and helping members to understand how macro-level system changes may impact their daily work. PAs play an important role in bridging the government’s health care vision and micro-level implementation.

### Theme #2: Negotiating with government

All participants described the importance of regular, formalized channels of communication with the government such as meetings or committees. The largest category supporting this theme is called “building trust and finding solutions”, which suggests the effective role of dynamic communication. Most participants emphasized the importance of taking initiative to build trusting relationships with government and to present themselves as solution-oriented.

“We don’t just complain, we actually come up with something that actually helps them do their job better. And the second thing is this trust, which is they haven’t seen us come out against them vociferously in the media, there have been no leaks, we are very respectful, they share decks, we never release it, we’ve never betrayed that trust.” (PA #8)

“I try and be as helpful as possible, I bring solutions…you want a reputation of bringing good solutions to the table being easy to work with. Not difficult at the civil servants level. And the politicians [saying], we need [them]…at the table.” (PA #7)

Regarding barriers, most participants referenced the issue of role clarity. They emphasized the importance of ensuring that their message reaches an appropriate receptor. This may be particularly difficult during times of transformation, when the government may reorganize or build new departments to lead the change. Locally, the government created a new agency, Ontario Health, to coordinate OHT development [22]. In some cases, PAs were unsure of who to communicate with, which necessitated building new relationships.

“We still don’t have clarity on exactly who’s responsible for what. We don’t get the sense that there’s clarity even within the government circles as to who should be dealing with what. There is definitely confusion…as to who we should be contacting regarding various issues.” (PA #2)

“A newer challenge for us was not knowing who the right receptor was. [We] have the same conversation five times with different groups, but it was sort of like we don’t really know, ultimately, who’s making the decision. So we just need to make sure that anyone who has a role in the decision has the information.” (PA #5)

The confusion related to communication carriers over to blurry vision for OHTs. Participants were supportive of the vision for integrated care but had little information regarding implementation.

“What is the vision of OHTs long term and of governance? I don’t think there’s a clear roadmap…that is probably [the] biggest hurdle as we’re encouraging and coaching our teams without a fallback or that vision to show them, sometimes it’s hard to get their buy in.” (PA #1)

“There’s a feeling that there is a whole bunch of policy people in government who spend their days thinking about implementation. There is not. They don’t realize that actually, [solutions must] come from the sector itself.” (PA #7)

The area where PAs emphasized their utility was in aligning the interests of their members with government policy. Here is an example of a PA enacting a connector role:

“These are the cases where we’re listening to our member needs. Yet, we’re also seeing the bigger picture. We’re trying to find compromises and solutions where we can push our teams in certain directions, but also be mindful of the fact that you can’t just keep asking teams to do more and more and more without funding. So it’s, you need to do A, B, and C and then we’ll consider, you know, making the requests for advocacy or advocating for additional investments.” (PA #1)

This theme describes the dynamic relationship between PAs and the government. All PAs engaged in regular communication with the government and valued these opportunities to maintain positive working relationships. However, times of system transformation often created significant communication barriers. PAs flagged challenges related to role clarity when communicating with multiple government departments and the lack of a detailed vision to guide the rollout of the health system transformation. To address this, PAs play a role beyond representing member needs, and bring forward practical solutions that support a variety of system stakeholders.

### Theme #3: Collaborating with stakeholders

A key function of PAs, as described by all participants, was collaboration with a wide variety of system stakeholders, including other PAs. The largest category within this theme described the utility of forming and/or participating in coalitions. Often, coalitions served as starting places for dialogue and opportunities to find alignment. Some participants emphasized the potential for amplifying a shared message during coalition discussions. Here are a few participant quotations on the topic of coalitions:

“We created one point of contact…so that the ministry does not have to reach out to different associations.” (PA #1)

“I’m a big leader of coalitions…if you’re a coalition builder, you have to be aware, not everything takes off and lands successfully. You have to have lots and lots of tries at it.” (PA #4)

Another large category was “finding alignment”, as common interests or priorities were described as providing a foundation to begin strategic discussions.

“At the end of the day, you look for the alignment. And you must be able to work with everybody to construct a vision that that is in alignment… working collaboratively to align with others, and sometimes very strange bedfellows that you align with.” (PA #4)

“It’s about building alignment around specific projects, and initiatives and figuring out where we agree and then we amplify. When we disagree, we negotiate, how will we deal with that? So rather than saying, let’s just be friends, we actually build relationships through the shared aims.” (PA #8)

Probing further into collaboration, participants shared the characteristics of a “good collaborator”, which revealed key concepts centring around mutual respect, trusting relationships and a commitment to improving the health care system:

“The people who are most successful in this role are not the bullies. And they’re not loud talkers. They’re not those that are banging on some drums somewhere, all by themselves. Ultimately, those people are not really part of the pack, if you want to put it that way. You have to you have to be very skilled at interpersonal relationships”. (PA #4)

“A good collaborator, there are two important characteristics a) openness, and b) trust. If you’re not open…you’re not open to hearing and negotiating, compromising, then it makes it much harder.” (PA #8)

Participants also identified a wide range of barriers to collaboration, including power imbalances, lack of resources, bureaucracy, lack of data, and negative historical relationships (e.g., turf wars, distrust of government and each other). Participants described the largest barrier as misalignment or different group interests:

“It’s just a fundamental difference in who we’re serving and who our membership is. Our membership is broad and spanning across all disciplines…as opposed to [PAs representing a single profession]. Yeah, so it is definitely different approaches.” (PA #1)

“One [barrier] is where the perspectives of the organizations really don’t mesh or we have a common perspective, that’s really hard to advance because of particular interests within a certain group… I think sometimes it’s about like, are we really diluting our message so much by partnering with another group or going so off message that it doesn’t make sense?” (PA #5)

When providing recommendations on how to improve collaboration between PAs, nearly all participants emphasized the need for a shared vision. Some participants suggested that developing a framework could be helpful to guide collaborative processes.

“You need a shared vision, which there isn’t one right now. I think you need collaborative governance. You need boards that are working together, which I think we don’t do enough of that right now.” (PA #1)

Some participants proposed that that it could be beneficial to increase their focus on serving the interests of the system, and ultimately patients.

“We need everybody to recognize the various roles that each can play. And it’s complimentary, you know, not competitive in providing patient health care…We can’t keep going down this road. We’ve been needing to have these mature conversations about health care for 20 years now. The biggest need in healthcare right now is just to say, how can we do things differently and improve patient care?” (PA #2)

“I think that if an association’s mission was around patients, and about really good health care policy, I think we would all collectively do better. It’s tricky.” (PA #7)

Participants were keen to reflect on their philosophies of collaboration and were able to provide many detailed examples. All PA philosophies were premised on the idea of “finding alignment”, as this provided a shared vision to work toward. Participating in formal communication structures, such as coalitions, allowed for dialogue with other groups to locate commonalities. Participants also highlighted the importance of attending to relationships and enacting principles of positive communication, such as trust and respect. A range of barriers to collaboration were identified, which highlighted differences in organizational resources, power, and historical relationships. These barriers, particularly a lack of resources, often resulted in different levels of stakeholder engagement. Regarding future recommendations to improve collaboration, participants suggested that prioritizing collective system needs could provide a starting place to find alignment.

### Theme #4: Reflecting on the PA role

Participants reflected on significant “shifts” or ways that their organization has evolved over time, often to improve collaboration with key stakeholders. Some participants described how (and why) their current collaborative strategies differ from historical relationships. Other participants described how their collaboration strategies shifted during uncertain times of transformation:

“[We have] evolved. I think as our board has evolved, it has caused us in our approach to evolve with our members as well and to be encouraging their boards to be less operational to be more strategic and more generative.” (PA #1)

“Why the shift? [We] bring a new sort of energy into it. I think that it’s also carving out a different role for [us] in some ways on these public policy issues. And I would say also, new research from our members that saying, we need to be heard here.” (PA #7)

The participants described how these “shifts” have helped them to continually reorient in a dynamic and crowded health policy space, which was particularly important during the pandemic. Although the focus of this research study is on context of purposeful health system transformation (such as OHT development), it is not possible to parse this from the unintended and simultaneous pandemic-related health system changes. Many participants described the unifying effects of COVID-19 on health care planning and delivery:

“Over COVID, [providers] were able to see the value of coming together, they were able to do a number of different important things that were helpful to them on the ground and their patients, early on… I think that helped demonstrate the value…as that was happening, we did a lot to highlight, profile and promote those good examples.” (PA #8)

“I think that [the pandemic] has been a catalyst for collaboration.” (PA #3)

However, a few participants reported that the pandemic slowed progress on OHT development:

“The development and the maturity of the OHTs has been stalled. In the beginning, people [were] getting interested…Seems like a good idea, then COVID hit, and it gave us a jumpstart to a higher level…and now, we’re starting to lose that momentum. And they need the next thing, not another pandemic. But the next thing to help them realize the benefits.” (PA #8)

“I think for most associations, OHTs have been put on the backburner [given] all of the COVID issues.” (PA #7)

This theme highlights the efforts of PAs to evolve in alignment with the changing nature of health care. The examples related to the COVID-19 pandemic clearly describe how PAs purposefully shift their strategies to manage emergent challenges.

## Discussion

This study has identified key functions enacted by PAs, particularly when influencing policy within the context of large-scale health system transformations. These functions describe a balancing act, undertaken by PAs to simultaneously attend to (1) supporting members, (2) negotiating with government, (3) collaborating with stakeholders, and (4) reflecting on the PA role. These findings extend recent work that highlights the sophisticated functions of PAs, such as medical associations [7]. Most peer-reviewed literature on the topic of PAs is profession-specific, particularly regarding medicine and nursing [7,23]. This study reports on the actions of diverse PAs, all involved with OHT development. Although we could not provide specific information of the PA characteristics, the PAs range considerably in size and membership.

The study findings reinforce the notion that PAs are well connected organizations that balance relationships between their members, government, and stakeholders. As mentioned, the study participants were senior level leaders, engaged in high level organizational discussions and with insight into strategic directions. The participants presented their PAs as deeply invested in the needs of their members and interested in learning about their concerns through surveys, committees, and other modes. In terms of member engagement, PAs played important roles in connecting members. This was a critical function during the early stages of OHT development, as it helped members to learn from the successes or struggles of others. PAs also engaged membership to help them understand how health system changes could impact their daily practices and helped them to access accurate information and educational resources. These findings help to further distinguish PAs from groups, such as unions, that tend to adopt a firm stance when advocating for member needs [24]. Rather, PAs play an important role of bringing forward solutions that address member concerns, especially in times of government driven health system transformations.

In this study, participants shared their reflections on the philosophies of their PAs, in relation to collaboration. Given the interconnected nature of PAs, there was a need to collaborate with stakeholders with various structures and degrees of power, including each other. This required interpersonal skills in communication, such as engaging in active listening and showing mutual respect; particularly when seeking to build trust in professional relationships. Many study participants described that collaboration starts by locating areas of alignment between PAs, as a shared vision provides a platform to amplify a shared message or advocate for a collective solution. The notion of collaboration appeared to be particularly important during times of health system transformation, as coalitions and partnerships served as effective strategies to amplify shared messages. These strategies may appear simple but can be difficult to enact in PAs given the historically secretive nature of their operations [7].

Our research team probed further to explore PA actions when alignment does not seem likely, often due to fundamental differences in member interests. Some participants suggested that it may be beneficial to consider collective or system needs. In a few interviews, there was discussion about whether the mandates of PAs should remain centred on the needs of the member, often a clinician. It was reinforced that seeking opportunities to support clinicians can help to improve patient care, during micro-level patient-provider interactions and macro-level health policy changes. Some local PAs have started to integrate system-focused language into newer iterations of their statements. This supports trends in international literature; wherein PAs are integrating concepts such as social determinants of health into their position statements and publications [25,26,27,28]. Reorienting the focus on patients and population health may serve as a facilitator for PA collaboration, especially when these principles usually underpin health system transformation.

The study findings related to how PAs negotiate with government describe the critical role that PAs in health system transformations. Study participants highlighted the variety of ways that their organizations have advocated for spaces that create opportunities for meaningful dialogue with various government departments. This type of communication has allowed many PAs to develop relationships in which they are viewed as credible, trustworthy and solution-oriented. PAs frequently helped the government to understand the perspectives of frontline clinicians and the realities of implementation that are linked to the transformative vision, which highlighted the critical ‘connector’ role that PAs enact during health system transformations. Often, the solutions generated by PAs support the government in determining how to implement their vision, in accordance with the needs of a variety of system users. The ‘connector’ function was clearly described in PAs with senior leadership who could leverage positive historical relationships and engage in authentic collaboration, underpinned by skills in deep listening and an ability to engage in dialogue to build shared aims.

The final theme described in this study sheds light on how and why PAs have evolved to become proficient in skills such as collaboration. Many participants described that a collaborative and relational approach often yields better quality solutions and implementation plans. In their current state, PAs appear to engage in regular reflection and are seeking to learn from members, government and other system stakeholders. Although a part of a crowded health policy environment, PAs enact overt strategies by demonstrating thought leadership through communications (e.g., media, publications) and covert leadership by proposing solutions in private negotiations with government and other stakeholders.

When viewing the study themes together, there are two main takeaways: (1) PAs play a critical role in the health ecosystem, primarily by virtue of their connections to the government, key stakeholders and members; (2) PAs influence large-scale health system transformations in a variety of ways. Understanding the important functions of PAs during health system transformations could support other stakeholders in realizing the potential value in collaborating with PAs, particularly during health system transformations.

## Limitations

Although we believe that the findings will be of interest to an international audience, we emphasize that this study was conducted in one province, Ontario and at one point in time. Additionally, most interviews were conducted with a single representative from their PA. Although the selected participant was knowledgeable about the contributions of their PA on the topic of OHTs, it is possible that the views and experiences of additional individuals were excluded. While the major themes are transferable, nuances regarding historical relationships with other stakeholders and the government may be different in health systems that differ significantly in structure from the Canadian context.

## Conclusion

The findings from this study describe the important and varied ways that PAs influence health system transformation. More importantly, this work adds to the limited scholarly literature on the roles, functions and philosophies of PAs. PAs are strategic, collaboration-oriented entities that play critical roles in supporting government to develop solutions to address the realities of healthcare delivery. Many of these solutions are informed by their deep engagement with members and interactions with other key stakeholders. Their functions related to leadership and influence are often hidden from public view, as communications with government and other stakeholders are usually private. This work highlights the value of consulting and collaborating with PAs, particularly within the context of health system transformations. Health services and policy researchers interested in understanding health system transformations may also use this study as a basis for including PAs in their future work. Future research could explore the factors that promote successful collaboration between PAs.

## Additional File

The additional file for this article can be found as follows:

10.5334/ijic.7017.s1 Appendix A. Interview Guide Questions.

## References

[1] Best A, Greenhalgh T, Lewis S, Saul JE, Carroll S, Bitz J. Large-system transformation in health care: a realist review. The Milbank Quarterly. 2012 Sep; 90(3): 421–56. DOI: 10.1111/j.1468-0009.2012.00670.x 22985277 PMC3479379

[2] Ferenchick EK, Rasanathan K, Polanco NT, Bornemisza O, Kelley E, Mangiaterra V. Scaling up integration of health services. The Lancet. 2018 Jan 13; 391(10116): 102–3. DOI: 10.1016/S0140-6736(18)30020-5 29353604

[3] Zonneveld N, Driessen N, Stüssgen RA, Minkman MM. Values of integrated care: a systematic review. International Journal of Integrated Care. 2018 Oct; 18(4): 1–12. DOI: 10.5334/ijic.4172 PMC6251066 30498405

[4] Miller R, Glasby J, Dickinson H. Integrated health and social care in England: ten years on. International Journal of Integrated Care. 2021 Oct; 21(4): 1–9. DOI: 10.5334/ijic.5666 PMC8555479 34754282

[5] Merton RK. The functions of the professional association. The American journal of nursing. 1958 Jan 1: 50–4. DOI: 10.2307/3461366 13487655

[6] American Medical Association. About. [webpage on the internet]. [cited 2023 March 15]. Available from: https://www.ama-assn.org/about.

[7] Brophy SA, Sriram V. Introduction to “recontextualizing physician associations: revisiting context, scope, methodology”. Journal of Health Politics, Policy and Law. 2021 Aug 1; 46(4): 641–52. DOI: 10.1215/03616878-8970852 33493296

[8] Indar A, Wright J, Nelson M. How do professional associations influence health system change? A critical literature review; 2022 (Unpublished).

[9] Erickson S. Health Professional Associations: Finding the Balance between Profession and Business. Health Industry Communication. 2011 Aug 3; 81.

[10] Embuldeniya G, Gutberg J, Sibbald SS, Wodchis WP. The beginnings of health system transformation: how Ontario Health Teams are implementing change in the context of uncertainty. Health Policy. 2021 Dec 1; 125(12): 1543–9. DOI: 10.1016/j.healthpol.2021.10.005 34702574

[11] Hughes G, Shaw SE, Greenhalgh T. Rethinking integrated care: a systematic hermeneutic review of the literature on integrated care strategies and concepts. The Milbank Quarterly. 2020 Jun; 98(2): 446–92. DOI: 10.1111/1468-0009.12459 32436330 PMC7296432

[12] Grudniewicz A, Tenbensel T, Evans JM, Gray CS, Baker GR, Wodchis WP. ‘Complexity-compatible’ policy for integrated care? Lessons from the implementation of Ontario’s Health Links. Social Science & Medicine. 2018 Feb 1; 198: 95–102. DOI: 10.1016/j.socscimed.2017.12.029 29310110

[13] Jiwani I, Fleury MJ. Divergent modes of integration: the Canadian way. International Journal of Integrated Care. 2011 Jan; 11(Special 10th Anniversary Edition): e018. DOI: 10.5334/ijic.578 21954371 PMC3180698

[14] Ontario Ministry of Health. Ontario Health Teams [Internet]. Toronto, ON: Queen’s Printer for Ontario; 2022 Jul 20. Available from: https://www.health.gov.on.ca/.

[15] Baker R, Blackstien-Hirsch P, Nelson M, Graham H, Sinclair L, Pinto A, et al. Governance and leadership challenges and strategies for Ontario Health Teams. International Journal of Integrated Care. 2022; 22(S2): 168. DOI: 10.5334/ijic.ICIC21256

[16] Bell B. Are Ontario Health Teams designed for failure? The Toronto Star [Internet]; 2019 Sep 3 [cited 2022 Oct 28]. Available from: https://www.thestar.com/opinion/contributors/2019/09/03/are-ontario-health-teams-designed-for-failure.html.

[17] Government of Ontario. Guidance for Ontario health teams: collaborative decision-making arrangements for a connected health care system; 2020 Jul. [cited 2023 15 Mar]. Available from: https://health.gov.on.ca/en/pro/programs/connectedcare/oht/docs/OHT_CDMA_Guidance_Doc.pdf.

[18] Detsky AS, Bogoch II. COVID-19 in Canada: experience and response. JAMA. 2020 Aug 25; 324(8): 743–4. DOI: 10.1001/jama.2020.14033 32790824

[19] McMahon M, Tamblyn R. The health system impact fellowship: perspectives from the program leads: comment on “CIHR health system impact fellows: reflections on ‘driving change’ within the health system”. International Journal of Health Policy and Management. 2019 Oct; 8(10): 623. DOI: 10.15171/ijhpm.2019.59 31657192 PMC6819624

[20] Sandelowski M. What’s in a name? Qualitative description revisited. Research in Nursing & Health. 2010 Feb; 33(1): 77–84. DOI: 10.1002/nur.20362 20014004

[21] Elo S, Kyngäs H. The qualitative content analysis process. Journal of Advanced Nursing. 2008 Apr; 62(1): 107–15. DOI: 10.1111/j.1365-2648.2007.04569.x 18352969

[22] Ontario Health. Our Story [Internet]. Toronto, ON: 2020 Jul 16. Available from: https://www.ontariohealth.ca/our-story.

[23] Simpson, CS, Velji, KA. Health policy advocacy: The role of professional associations. Kingston: Queen’s University, The Monieson Centre; 2015 [2023 15 Mar]. Available from: https://healthcare.report/Resources/Whitepapers/6e7a4f19-1479-45d0-b345-89bb43d91f6d_2015_QHPCC_Whitepaper.pdf#page=67.

[24] Gilchrist JM. Profession or union: who will call the shots? Canadian Journal of Nursing Research Archive. 1969 Apr 13: 4–10. 5201158

[25] Avni S, Filc D, Davidovitch N. The Israeli Medical Association’s discourse on health inequity. Social Science & Medicine. 2015 Nov 1; 144: 119–26. DOI: 10.1016/j.socscimed.2015.09.015 26409421

[26] Brophy SA. Health or politics? *Organizational maintenance in the AAFP*. Journal of Health Politics, Policy and Law. 2019 Feb 1; 44(1): 43–66. DOI: 10.1215/03616878-7206719

[27] McKinlay JB, Marceau LD. The end of the golden age of doctoring. International Journal of Health Services. 2002 Apr; 32(2): 379–416. DOI: 10.2190/JL1D-21BG-PK2N-J0KD 12067037

[28] Stevens RA. Public roles for the medical profession in the United States: beyond theories of decline and fall. The Milbank Quarterly. 2001 Sep; 79(3): 327–53. DOI: 10.1111/1468-0009.00211 11565160 PMC2751200

