# Supplementary material for: Exploring how Professional Associations Influence Health System Transformation: The Case of Ontario Health Teams
Source: Int J Integr Care. 2023 May 24;23(2):19. doi: 10.5334/ijic.7017 (PMC10215991; doi:10.5334/ijic.7017)
Supplement: Appendix A. — Interview Guide Questions. [file ijic-23-2-7017-s1.pdf]

Appendix A
Interview Guide Questions

The first few questions pertain to your role in your organization.

- What are your responsibilities in your current role?
- How do you characterize the overarching purpose/mission of the [specific professional association]?
    - How does your association's structure support the functions that you have described?

From the literature, we know that there it can be challenging for professional associations to navigate issues where member needs are not aligned with system needs.

- Reflecting on this, can you describe a recent example of when your association needed to balance (or align) your member needs with system priorities? Can you provide an example related to the topic of OHTs?
    - How has your organization reconciled the gaps between member interests and system priorities?
    - Do you have any specific processes or frameworks to assist you with these decisions?
    - Are there instances where it may be necessary to portray different messages internally versus externally (i.e., to the public)?
    - Can you provide an example of when you have had to manage a message differently internally versus publicly?

Professional associations a have a responsibility to their members and often work to influence system transformation in ways that align with their needs.

- Generally (not specific to OHTs), what strategies do you use to influence system transformation or change (e.g., communications, policy, GR, legal, stakeholder relations etc.)?
- What strategies have you used to within the context of OHT development?
    - Who is the intended audience for these strategies?
    - What resources do these strategies require?
    - What functions (or functional areas) have you expanded or prioritized to implement these strategies?
    - Do you have a generally consistent approach to issues?

- Where do you wish you had greater capacity? Are there certain areas that would be helpful to expand

OHT development is a complex process that started in 2019 and continues to be impacted significantly by the pandemic.

- What strategies did you use to influence OHT development initially and why? Who were your efforts targeted toward?

- How have you adjusted your strategies over time (perhaps in response to evolving challenges)? Has this required your organization to expand, build or shift capacity in some areas?
- What strategies are you currently focusing and why? Who are you communicating and/or collaborating with most often?

Stakeholder Collaboration

- Regarding collaboration, who (or which organizations) do you generally tend to collaboration with and why?
- Thinking about OHT development, which collaborators have been most helpful and why?
    - What makes a good collaborator (e.g., similar approach, structure or interests/values)?
    - Prompt (if needed): Which groups tend to share your interests?
- Are there any examples of organizations/groups that you have built new relationships with, to strategically amplify your influence on OHT development?
- Who have you had difficulty collaborating with and why?
- Do you have recommendations on how to improve collaboration between stakeholders (e.g., other professional associations, regulators, national bodies, broader system stakeholders)?
- Do you see any opportunities to leverage common areas of alignment that have been unexplored?

Government Relations

- Generally, how would you characterize your working relationship with the government?
- Thinking specifically about OHT development, how have you been able to work with the government? What strategies are you using to communicate? How successful do you think these strategies have been?
- Do you have any recommendations to improve how the government manages relationships with their stakeholders?
- Can you give me an example of when your interests were in conflict with the government? How did you manage this?

Are there any other stakeholder (e.g., professional associations) that we should interview on this topic?

Is there any additional information that you would like to share?
